# Correlations between Phlegm Syndrome of Chinese Medicine and Coronary Angiography: A Systematic Review and Meta-Analysis

**DOI:** 10.1155/2015/751743

**Published:** 2015-06-09

**Authors:** Qiu-Yan Zhang, Hao Liang, Hou-Wu Gong, Hui-yong Huang, Xiao-qing Zhou, Xiang Sun

**Affiliations:** ^1^Hunan University of Chinese Medicine, Science-Education Industrial Park, Yuelu Region, Changsha 410208, China; ^2^The First Hospital of Hunan University of Chinese Medicine, No. 95 of Shaoshan Road, Yuhua Region, Changsha 410007, China; ^3^No. 8 Hospital of Changsha, No. 22 of Xingsha Street, Xingsha Region, Changsha 410100, China

## Abstract

Phlegm is one of the most common patterns of coronary artery disease (CAD) in Chinese medicine. Our research was aimed at investigating the association between phlegm syndrome of CAD and coronary angiography (CAG) by meta-analysis. According to inclusion criteria, a total of 30 studies involving 5,055 CAD patients were included. The meta-analysis showed that phlegm syndrome patients were prone to multivessel disease (28 studies, OR = 1.53, 95% CI, 1.24 to 1.88, *P* < 0.01) and higher Gensini score (2 studies, OR = 5.90, 95% CI, 1.86 to 9.94, *P* = 0.004), but not obviously relevant to severe stenosis (≥75%) of coronary arteries (13 studies, OR = 1.20, 95% CI, 0.63 to 2.27, *P* = 0.57). We concluded that the coronary arteries lesions of CAD patients with phlegm syndrome were more severe than those with nonphlegm syndromes. Phlegm syndrome should, therefore, be regarded as a dangerous pattern of CAD with worse prognosis.

## 1. Introduction

Coronary artery disease (CAD) is a prevalent disease against human health, dedicated to one of the leading causes of death [1]. Traditional Chinese medicine (TCM) has fought against CAD (belonging to the categories of Xiong-bi and cardialgia in TCM) for thousands of years, establishing unique theories for etiology and systems of diagnosis and treatment. The syndrome (also called pattern [2]) and syndrome differentiation are the comprehensive analysis of clinical information and can be deemed to be the TCM theoretical interpretation of the symptom profiles [3]. Syndrome differentiation can be used for further stratification of the patients' conditions with certain disease, identified by orthodox medical diagnosis. It guides the choice of treatment either by acupuncture or by TCM herbal formulae and helps in the improvement of efficacy of the selected intervention [4].

Phlegm and blood-stasis are regarded as the most common patterns of CAD patients [5]. They are significantly related to hyperlipidemia [6] and atherosclerosis and platelet activating system [7], which determines the prognosis and stratification of CAD. Phlegm is defined as a viscous, turbid pathological product that can accumulate in the body, causing a variety of diseases [2]. TCM has two general categories of phlegm: broadly defined phlegm and narrowly defined phlegm (visible phlegm such as nasal discharge or sputum from respiratory passages). If the former, invisible phlegm accumulated in the chest and blocked the channels and vessels of heart, it would lead to the so-called phlegm syndrome of Xiong-bi characterized as choking or crushing chest discomfort, shortness of breath, heavy feeling in the limbs, gastric stuffiness, sticky slimy sensation in the mouth, slimy and thick tongue coating, and slippery pulse [8], which was similar to manifestations of typical angina.

Lots of studies about the correlations between TCM syndromes and coronary angiography (CAG) were published recently, providing new objective information for syndrome differentiation of CAD [9]. Our previous pooled analysis showed a strong relation between blood-stasis syndrome and CAG [10], but the relevance of phlegm syndrome to presentations of CAG was not explored. This study was to investigate whether the phlegm syndrome was related to CAG through a systematic review and meta-analysis.

## 2. Methods

### 2.1. Search Strategy

MOOSE (Meta-Analysis of Observational Studies in Epidemiology) and PRISMA (Preferred Reporting Items for Systematic Reviews and Meta-Analyses) statements were carefully consulted [41, 42]. To identify all relevant studies, we performed a literature search (Chinese and English languages) in China Academic Journal Network Publishing Database (CAJD), Chinese Biomedical Literature Database (CBM), China Doctor Dissertation Full-Text Database (CDFD), Chinese Selected Master's Theses Full-Text Databases (CMFD), and Medline and Embase (January 1990 through June 2014) under strict-making search strategy using Medical Subject Heading (MeSH) term (list of tables, Table 1). We also hand-searched the reference lists of all primary studies and reviews identified by the initial search.

### 2.2. Study Selection

All diagnostic cross-sectional studies, cohort studies, case-control studies, and randomized studies were retrieved to investigate association between TCM syndromes and presentations of CAG. We included a study if (1) obstructive CAD, with ≥50% diameter stenosis, was selected as the standard for significant CAD, using catheter-based X-ray angiography as the compared standard; (2) reported cases are in absolute numbers that can distinguish between phlegm syndrome and the others; (3) CAG results included at least one of the following parameters: the number of diseased arteries (defined* multivessel disease* [43] as cases with more than one stenotic (≥50%) diameter coronary artery), degree of coronary artery stenosis (defined* severe artery stenosis* as ≥75% by CAG), and Gensini score [44] (a common score system for evaluating the culprit coronary vessels). Studies were excluded if (1) animals; (2) review and case report; (3) study focused on specific CAD population, for example, female CAD patients, acute myocardial infarction, CAD with diabetes, and so on (4) duplicate reports.

### 2.3. Data Extraction and Quality Assessment

Two independent reviewers (Liang and Sun) extracted the following data from the selected studies. Inconsistencies were settled by discussion and consensus. We extracted year of publication, study design, clinical setting, methods of syndrome differentiation, and angiographic parameters. Methodological quality of included primary studies was assessed by two authors (Zhou and Gong) using a modified QUADAS-2 tool that included eight items [45]:* patient selection*: (1) consecutive or randomized patients; (2) avoiding inappropriate exclusions; (3) inclusion criteria of patients described;* syndrome differentiation*: (4) syndrome differentiation results interpreted without knowledge of the results of CAG; (5) syndrome differentiation by more than two independent doctors;* reference standard*: (6) CAG results interpreted without knowledge of the results of the syndrome differentiation;* flow and timing*: (7) an appropriate interval between syndrome differentiation and CAG; (8) perspective design. They are answered as “yes,” “no,” or “unclear” and are phrased such that “yes” indicates low risk of bias. Tabular and graphic displays served in summarizing quality assessments.

### 2.4. Statistical Analysis and Data Synthesis

To identify potential correlations between phlegm syndrome and CAG, we calculated an overall OR with a fixed or random effects model meta-analysis for the indices, which assumes the underlying effect varies according to studies. We performed tests of heterogeneity between studies using a standard chi-square test and *I* ^2^ statistic [46]. To examine sources of heterogeneity, subgroups of studies identified by selected covariates were meta-analyzed separately. Egger's test and Begg's funnel plot were applied for detecting publication bias in the meta-analysis. One-way sensitivity analysis was performed to assess the stability of the results; namely, a single study in the meta-analysis was deleted each time to reflect the influence of the individual data set to the pooled OR [47]. Statistical significance was set at *P* < 0.05 and all statistical analyses were performed using RevMan 5.2.2 (The Cochrane Collaboration).

## 3. Results

### 3.1. Description of Included Studies

**Figure 1:**
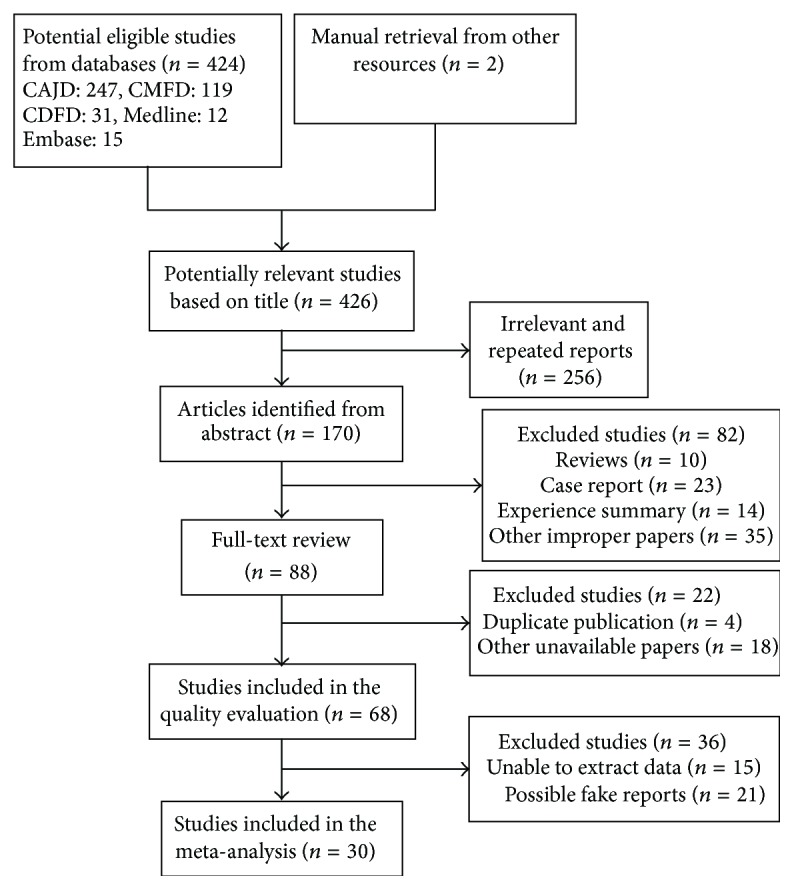
Flow diagram of studies considered for the review.

The literature process was outlined in Figure 1, and 88 potentially relevant studies were retrieved for detailed evaluation. 58 were excluded because (1) they had overlapping data; (2) it was not possible to calculate absolute numbers from the presented data; (3) or data was inconsistent in the context of the article. Finally, 30 included studies met the inclusion criteria, 29 Chinese articles, and 1 English article. All of the studies were from hospitals of China. Study and population characteristics of the included studies are summarized in Table 2, involving a total of 5,029 patients, reporting age ranging from 37 to 87 years. Analysis of the syndromes showed that there were 1,674 (33.3%) phlegm syndrome patients compared with 3,355 (66.7%) of the others. Most studies reported elective assessment for CAD, with study groups including those with suspected and known CAD. Eight studies were theses from medical universities of China. Methods for syndrome differentiation of CAD were divided into syndrome-element differentiation (SED) and other methods called conventional syndrome differentiation (CSD). CSD contains the classical syndrome differentiation methods, such as viscera syndrome differentiation and eight-principal syndrome differentiation [2].

### 3.2. Quality Assessment of Included Studies

**Figure 2:**
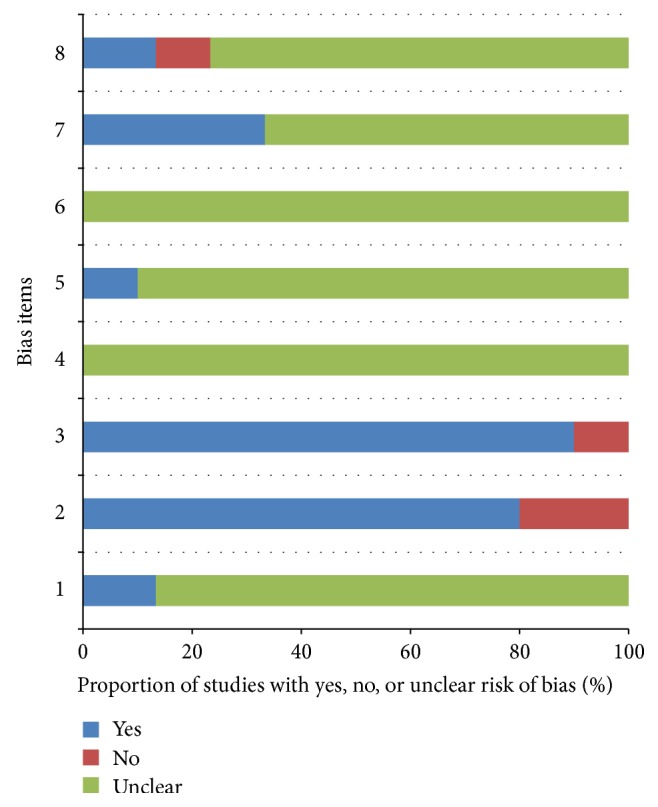
Graphic display of quality assessment. Note: (1) consecutive or randomized patients; (2) avoiding inappropriate exclusions; (3) inclusion criteria of patients described; (4) syndrome differentiation results interpreted without knowledge of the results of CAG; (5) syndrome differentiation by more than two independent doctors; (6) CAG results interpreted without knowledge of the results of the syndrome differentiation; (7) an appropriate interval between syndrome differentiation and CAG; (8) perspective design.

Table 3 and Figure 2 summarized the quality assessment for the 30 full-text studies. Quality assessment of most studies was not satisfactory, especially the blinding method. None of the studies for those interpreting syndrome differentiation data were blinded to the results of the reference standard test (test review bias avoided) and vice versa (diagnostic review bias avoided).

### 3.3. Results of Meta-Analysis

**Figure 3:**
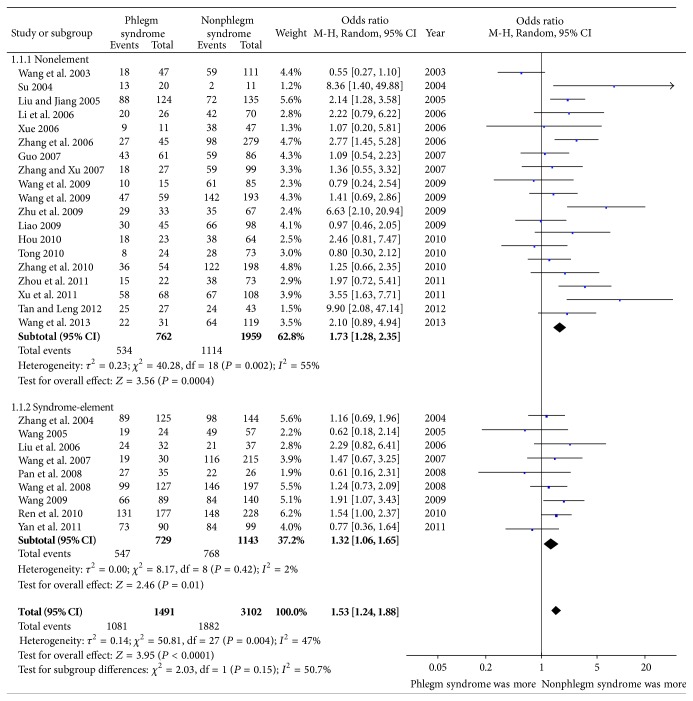
Meta-analysis of association between phlegm syndrome and multivessel disease.

**Figure 4:**
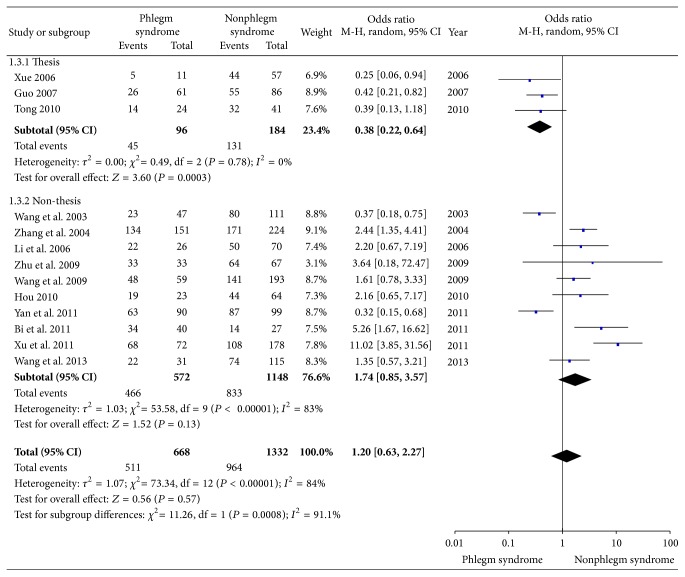
Meta-analysis of association between phlegm syndrome and severe artery stenosis.

**Figure 5:**
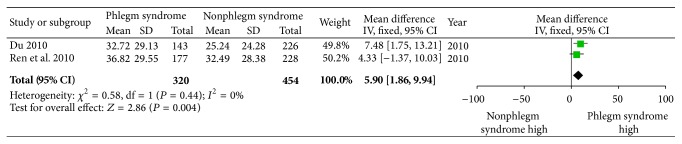
Meta-analysis of association between phlegm syndrome and Gensini score.

After the 30 eligible studies involving 5,055 CAD patients were pooled, there was a significant association between phlegm syndrome and CAG in some parameters. Overall, the multivessel disease OR associated with phlegm syndrome was 1.53 (95% CI, 1.24 to 1.88, *P* < 0.01; *I* ^2^ = 47%, Figure 3), while severe artery stenosis was 1.20 (95% CI, 0.63 to 2.27, *P* = 0.57; *I* ^2^ = 84%, Figure 4) and Gensini score was 5.90 (95% CI, 1.86 to 9.94, *P* = 0.004; *I* ^2^ = 0%, Figure 5). Although the heterogeneity among the analyzed studies was high, most of the studies referred to a positive association for multivessel disease. As the studies were divided into two subgroups by SED or CSD, the OR of multivessel disease in SED group was 1.34 (95% CI, 1.08 to 1.67, *P* = 0.008; *I* ^2^ = 10%, Figure 3), showing that the heterogeneity between the studies decreased substantially compared with the main analysis (Figure 3). For the association between phlegm syndrome and degree of artery stenosis, though the result was negative, the heterogeneity between the analyzed studies of thesis subset sharply reduced (*I* ^2^ = 0) compared with the main analysis (Figure 4).

### 3.4. Results of Sensitivity Analysis and Publication Bias

**Figure 6:**
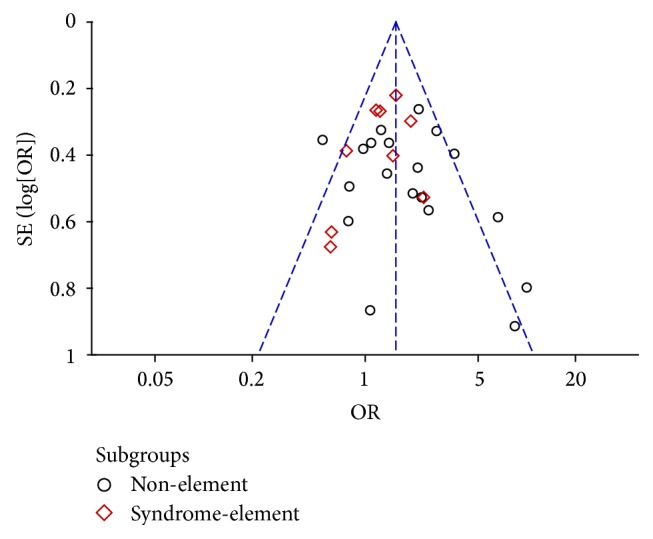
Funnel plot for publication bias by association between phlegm syndrome and multivessel disease.

No individual studies influencing the summary OR found by one-way sensitivity analysis, indicating that the pooled data was stable. There was no evidence of publication bias, because the funnel plot according to meta-analysis of association between phlegm syndrome and multivessel disease did not show obvious asymmetry by visual inspection (Figure 6), which was also confirmed by Egger's test (*P* = 0.527). However, some studies located out of the border, which may affect the result of the meta-analysis.

## 4. Discussion

This was the first meta-analysis to address the relationship between phlegm syndrome and CAG. The parameters of culprit vessel, stenosis degree, and Gensini score under CAG were important indicators for CAD patients, and we discovered that serious coronary artery lesions are associated with the presence of phlegm syndrome. The phlegm syndrome patients were inclined to suffer from multivessel disease and higher Gensini score, but no obvious correlation with the severe artery stenosis was found, which was different from our previous study of meta-analysis for the blood-stasis syndrome [10]. The difference can partly explain the essence of phlegm and blood-stasis syndrome. Blood-stasis and phlegm are both pathological products of abnormal metabolism of body fluid and blood in Chinese medicine. Phlegm formed in the spleen can easily expand and accumulate not only in the lungs but also in almost all other parts of the body, including five viscera, six bowels, joints, and surface tissue, through Meridians or Triple Energizers [48, 49]. Multivessel disease could be considered as diffuse changes of coronary arteries representing the pathological features of phlegm. The blood-stasis focused on the “stasis,” which means block, retardance, and fixation, so patients of blood-stasis pattern were significantly related to >75% degree of artery stenosis [10]. Unfortunately, we were unable to judge which syndrome was worst (phlegm or blood-stasis) for difficulty in extracting the independent data.

Considered as the base of modernization and scientification of TCM, the syndrome diagnostic criteria began to be researched from the 1980s. They were drawn up by different organizations and departments nonrecognized by one another, including Tentative Standard approved by National Academic Seminar on TCM Syndrome Differentiation Based Treatment of CAD in 1980 and Standards for Diagnosis and Curative Effect of TCM Diseases and Syndromes by State Administration of Traditional Chinese Medicine in 1994. Even the process of syndrome differentiation was judged by symptoms and signs with accepted criterion. Plus, the results could be affected by the doctor's learning, medical experience, academic origins, methods, and other factors. Therefore, the judgment made by doctors on cause, location, nature, and trend of disease is individual and variable, and no wonder that 10 TCM doctors may give 10 different diagnoses when facing the same patient [50]. It is crucial, however, to find the source of heterogeneity, usually an obstacle to the meta-analysis. Based on our subgroup analysis, it is strongly supported that different diagnostic methods were the source of heterogeneity and restriction for syndrome differentiation. The heterogeneity of syndrome differentiation based on SED studies decreased to 2% (Figure 3), while the CSD group increased a little. SED method, now widely used in clinical and basic research, was created by Professor Zhu et al. [51], who proposed the theory of syndrome-element and divided those “elements” into categories of location and nature. The differentiation system was established by clarifying that “syndrome” was composed of the key elements of the location and the nature of disease. Thus, a complete syndrome name was combined by elements of location and nature diagnosed from symptoms and signs. Unlike inflexible CSD, the syndrome could be free combination of possible syndrome-elements, allowing any forms of elements summation. For instance, phlegm is its high affinity with other pathogenic factors such as heat, cold, wind, dampness, and dryness and, because of this, phlegm is often combined with other pathogenic factors to form more complex patterns such as heat-phlegm, cold-phlegm, or wind-phlegm [52]. Moreover, our subanalysis proved that SED method led to homogeneity between studies, which would be more promising than CSD. The heterogeneity was also affected by the factor of thesis or not. For postgraduates lack of clinical experience, their diagnosis of syndrome differentiation may be different from older doctors, causing a discrepancy of studies. In a word, the thinking characteristics of syndrome are more similar to physical image thinking rather than image thinking [50]; thus, methods may play an important role during implementation based on the syndrome differentiation criteria.

Any process that yields information used to inform patient management can be regarded as a clinical test [53]. The basic aim of test accuracy studies is to assess how well a test can distinguish between people with and without the disease/syndrome/condition of interest [54]. Nevertheless, unlike index test compared with a reference standard in western medicine, the diagnostic test accuracy (DTA), such as specificity or sensitivity, was still tough for syndrome differentiation just by a profile of symptom combination, or clinical phenotypes. Above all, the design of syndrome differentiation research is similar to diagnostic tests, which means cross-sectional and descriptive in nature [55], and RCT [56, 57] case-control and two-gate designs could be feasible [55]. Using QUADAS-2 tool for quality assessment was an innovation of our research. Avoiding subjective impact on researchers, double blind, for instance, is the key to quality of diagnostic study. In the summary of tabular and graphic results, items of (4), (5), and (6) concerning improving objective diagnosis for syndromes were not optimistic (Table 3, Figure 2). It tells us that most of TCM researchers did not take diagnostic design seriously. Lack of blinding can lead to overestimation of test accuracy, especially when the interpretation of test results is subjective [58].

Evaluation score systems of CAG, such as Gensini score [44] and SYNTAX score [59], are more comprehensive and better than just number or degree of stenosis for vessel lesions to estimate conditions of diseased coronary arteries. Due to difficulty of extracting available data, only two studies containing Gensini score were included in the meta-analysis. Moreover, CAD patients could be subdivided into acute coronary syndrome (ACS) and non-ACS, and ACS attacks suddenly often with worse outcomes than non-ACS. The clinical manifestations and CAG of the two types of patients are quite different [60]. Once mixed up, it would produce more difficulties and confusion for the study. We hope patient spectrum of next researches is aimed at ACS or non-ACS individually. Last but not least, there were no standards or statements specific for publishing meta-analysis of diagnostic tests, so as the TCM syndrome diagnosis. The researchers have to only reference other standards, such as MOOSE [61] to finish the review, to some degree limiting the diagnostic research on meta-analysis.

## 5. Conclusions

From the meta-analysis, we found there were correlations between phlegm syndrome of CAD patients and image of CAG: the coronary arteries lesions of CAD patients with phlegm syndrome were more severe than those with other syndromes. Syndrome differentiation was not only important for diagnosis and treatment, but also useful for the prognosis. Phlegm syndrome could be considered as risk syndrome of CAD patients, whom doctors should pay closer attention to. However, from the quality assessment, we found that the design quality of TCM diagnostic test urgently needs to be addressed.

## Figures and Tables

**Table 1:** Search strategy.

Number	Search terms	Detailed strategy in CAJD and Medline	
		CAJD (Chinese)	Medline (English)
#1	Coronary heart disease (冠心病) or coronary artery disease	(SU = (“冠心病” + “冠状动脉粥样硬化性心脏病”) or TI = (“冠心病” + “冠状动脉粥样硬化性心脏病”) or KY = (“冠心病” + “冠状动脉粥样硬化性心脏病”)) and (SU = (“中医” + “证型” + “辨证”) or TI = (“中医” + “证型” + “辨证”) or KY = (“中医” + “证型” + “辨证”)) and (SU = (“冠脉造影” + “冠状动脉造影”) or TI = (“冠脉造影” + “冠状动脉造影”) or KY = (“冠脉造影” + “冠状动脉造影”))	(Chinese medicine [Title/Abstract]) AND (Coronary angiography [Title/Abstract]) AND (syndrome [Title/Abstract] OR pattern [Title/Abstract])
#2	Xiong-bi (胸痹) or chest pain (心痛)		
#3	Chinese medicine (中医) or syndrome (证) or pattern		
#4	Coronary angiography (冠状动脉造影) or CAG (冠脉造影)		
#5	Search #1 or #2		
#6	Search (#1 or #2) and #3		
#7	Search (#1 or #2) and #3 and #4	Number of papers: 245	Number of papers: 12

**Table 2:** Study and population characteristics of the included studies.

Number	Author, year	Age	*n*	Male/female	Thesis	Clinical setting	Method of syndrome differentiation	Syndrome overlap	Indexes of CAG
1	Wang et al., 2003 [11]	59.2 ± 7.8	158	98/60	No	Cross-sectional	CSD	No	CVN, DAS (70% as the classification)
2	Su, 2004 [12]	69.1 (unknown SD)	31	Unknown	Yes	Case-control	CSD	No	CVN
3	Zhang et al., 2004 [13]	58.5 ± 10.2	269	Unknown	No	Case-control	SED	Yes	CVN
4	Liuand Jiang, 2005 [14]	63.8 ± 9.6	259	209/50	No	Cross-sectional	CSD	No	CVN, Gensini score
5	Wang, 2005 [15]	64.6 ± 11.5	81	52/29	Yes	Cross-sectional	SED	Yes	CVN
6	Li et al., 2006 [16]	59.6 ± 10.1	96	77/19	No	Cross-sectional	CSD	No	CVN, DAS, Gensini score
7	Liu et al., 2006 [17]	65.4 ± 10.8	69	52/61	No	Cross-sectional	SED	Yes	CVN
8	Xue, 2006 [18]	Unknown	68	37/31	Yes	Cross-sectional	CSD	No	CVN, DAS, Gensini score
9	Zhang et al., 2006 [19]	57.6 (unknown SD)	279	—	No	Case-control	CSD	No	CVN
10	Guo, 2007 [20]	Unknown	147	104/43	Yes	Cross-sectional	CSD	No	CVN, DAS
11	Wang et al., 2007 [21]	Unknown	81	Unknown	No	Case-control	SED	Yes	CVN
12	Zhangand Xu, 2007 [22]	59.8 ± 12.3	126	99/27	No	Cross-sectional	CSD	No	CVN, DAS
13	Pan et al., 2008 [23]	68.0 ± 10.7	61	40/21	No	Cross-sectional	SED	Yes	CVN
14	Wang et al., 2008 [24]	58.2 ± 10.58	324	250/74	No	Cross-sectional	SED	Yes	CVN
15	Liao, 2009 [25]	Unknown	143	68/75	Yes	Cross-sectional	CSD	No	CVN, DAS
16	Wang, 2009 [26]	Unknown	100	57/43	Yes	Cross-sectional	CSD	No	CVN, Gensini score
17	Wang et al., 2009 [27]	Unknown	252	192/60	No	Cross-sectional	CSD	No	CVN, DAS
18	Wang et al., 2009 [28]	63.4 ± 11.7	229	128/101	No	Cross-sectional	SED	Yes	CVN, Gensini score
19	Zhu et al., 2009 [29]	Unknown	100	85/15	No	Cross-sectional	CSD	No	CVN, DAS
20	Du, 2010 [30]	59.6 ± 11.2	369	291/78	Yes	Cross-sectional	SED	Yes	Gensini score
21	Hou, 2010 [31]	Unknown	87	48/39	No	Cross-sectional	CSD	No	CVN, DAS, Gensini score
22	Ren et al., 2010 [32]	58.3 ± 10.7	405	307/98	No	Cross-sectional	SED	Yes	CVN, Gensini score
23	Tong, 2010 [33]	62.8 ± 9.3	97	57/40	Yes	Cross-sectional	CSD	No	CVN, DAS
24	Zhang et al., 2010 [34]	59.8 ± 12.3	368	252/116	No	Cross-sectional	CSD	No	CVN, DAS (70% as the classification), Gensini score
25	Bi et al., 2011 [35]	67.2 ± 8.3	67	Unknown	No	Cross-sectional	SED	Yes	DAS
26	Xu et al., 2011 [36]	59. 6 ± 10.1	250	200/50	No	Cross-sectional	CSD	No	CVN, Gensini score
27	Yan et al., 2011 [37]	69.0 ± 10.5	189	103/86	No	Cross-sectional	SED	Yes	CVN, DAS
28	Zhou et al., 2011 [38]	Unknown	95	73/22	No	Cross-sectional	CSD	No	CVN, DAS, Gensini score
29	Tanand Leng, 2012 [39]	Unknown	70	38/32	No	Cross-sectional	CSD	No	CVN
30	Wang et al., 2013 [40]	Unknown	150	68/82	No	Cross-sectional	CSD	No	CVN

		Total	5055						

Note: CSD = conventional syndrome differentiation, CVN = culprit-vessel number, DAS = degree of artery stenosis, and SED = syndrome-element differentiation.

**Table 3:** Tabular display of quality assessment.

Number	Study, year	Patient selection			Syndrome differentiation		Reference standard	Flow and timing	
		(1)	(2)	(3)	(4)	(5)	(6)	(7)	(8)
1	Wang et al., 2003 [11]	Unclear	Yes	Yes	Unclear	Unclear	Unclear	Yes	Unclear
2	Su, 2004 [12]	Unclear	Yes	Yes	Unclear	Unclear	Unclear	Yes	Unclear
3	Zhang et al., 2004 [13]	Yes	Yes	Yes	Unclear	Unclear	Unclear	Yes	Yes
4	Liu and Jiang, 2005 [14]	Unclear	No	Yes	Unclear	Unclear	Unclear	Unclear	Unclear
5	Wang, 2005 [15]	Yes	Yes	Yes	Unclear	Unclear	Unclear	Unclear	Unclear
6	Li et al., 2006 [16]	Unclear	Yes	Yes	Unclear	Unclear	Unclear	Unclear	Unclear
7	Liu et al., 2006 [17]	Unclear	Yes	Yes	Unclear	Unclear	Unclear	Yes	Unclear
8	Xue, 2006 [18]	Unclear	Yes	Yes	Unclear	Yes	Unclear	Unclear	Unclear
9	Zhang et al., 2006 [19]	Unclear	Yes	Yes	Unclear	Yes	Unclear	Yes	Yes
10	Guo, 2007 [20]	Unclear	No	Yes	Unclear	Unclear	Unclear	Unclear	Unclear
11	Wang et al., 2007 [21]	Unclear	No	Yes	Unclear	Unclear	Unclear	Yes	Yes
12	Zhang and Xu, 2007 [22]	Unclear	No	No	Unclear	Unclear	Unclear	Unclear	Unclear
13	Pan et al., 2008 [23]	Unclear	Yes	Yes	Unclear	Unclear	Unclear	Yes	Unclear
14	Wang et al., 2008 [24]	Unclear	Yes	Yes	Unclear	Unclear	Unclear	Yes	Unclear
15	Liao, 2009 [25]	Yes	No	Yes	Unclear	Unclear	Unclear	Yes	No
16	Wang, 2009 [26]	Unclear	Yes	Yes	Unclear	Yes	Unclear	Unclear	Unclear
17	Wang et al., 2009 [27]	Unclear	Yes	Yes	Unclear	Unclear	Unclear	Unclear	Yes
18	Wang et al., 2009 [28]	Unclear	Yes	Yes	Unclear	Unclear	Unclear	Unclear	Unclear
19	Zhu et al., 2009 [29]	Unclear	Yes	Yes	Unclear	Unclear	Unclear	Unclear	Unclear
20	Du, 2010 [30]	Unclear	Yes	Yes	Unclear	Unclear	Unclear	Yes	No
21	Hou, 2010 [31]	Unclear	Yes	No	Unclear	Unclear	Unclear	Unclear	Unclear
22	Ren et al., 2010 [32]	Unclear	Yes	Yes	Unclear	Unclear	Unclear	Unclear	Unclear
23	Tong, 2010 [33]	Unclear	Yes	Yes	Unclear	Unclear	Unclear	Unclear	No
24	Zhang et al., 2010 [34]	Unclear	No	No	Unclear	Unclear	Unclear	Unclear	Unclear
25	Bi et al., 2011 [35]	Yes	Yes	Yes	Unclear	Unclear	Unclear	Unclear	Unclear
26	Xu et al., 2011 [36]	Unclear	Yes	Yes	Unclear	Unclear	Unclear	Unclear	Unclear
27	Yan et al., 2011 [37]	Unclear	Yes	Yes	Unclear	Unclear	Unclear	Unclear	Unclear
28	Zhou et al., 2011 [38]	Unclear	Yes	Yes	Unclear	Unclear	Unclear	Unclear	Unclear
29	Tan and Leng, 2012 [39]	Unclear	Yes	Yes	Unclear	Unclear	Unclear	Unclear	Unclear
30	Wang et al., 2013 [40]	Unclear	Yes	Yes	Unclear	Unclear	Unclear	Unclear	Unclear

Note: (1) consecutive or randomized patients; (2) avoiding inappropriate exclusions; (3) inclusion criteria of patients described; syndrome differentiation: (4) syndrome differentiation results interpreted without knowledge of the results of CAG; (5) syndrome differentiation by more than two independent doctors; (6) CAG results interpreted without knowledge of the results of the syndrome differentiation; (7) an appropriate interval between syndrome differentiation and CAG; (8) perspective design.
